# Correlation of Dentistry and the Practice of Medicine*Read before the Virginia State Dental Association in Richmond, Va., and reprinted from the Virginia Medical Monthly, June, 1923.

**Published:** 1923-10

**Authors:** Manfred Call

**Affiliations:** Richmond, Va., Dean and Professor of Clinical Medicine, Medical College of Virginia


### CORRELATION OF DENTISTRY AND THE PRACTICE OF MEDICINE *

BY MANFRED CALL, M. D., RICHMOND, VA.

Dean and Professor of Clinical Medicine, Medical College of Virginia.

The opportunity to appear before an association of the dignity and importance of the Virginia State Dental Association is an honor to be coveted, and I wish to convey to you this acknowledgment of my appreciation of your invitation to read before you a paper on a subject of interest to the allied professions of Medicine and Dentistry.

---

* Read before the Virginia State Dental Association in Richmond, Va., and reprinted from the Virginia Medical Monthly, June, 1923.

It would be presumptuous for me, granting that I had the technical knowledge, to attempt to enlighten you on the fundamentals of your own profession; nor can I attempt, as a representative of the medical profession, to bring to you the consensus of opinion on the Correlation of Medical and Dental Practice. While this interdependence has been recognized in the abstract for some time, an appreciation of this relationship is now of increasing importance if we are to reach an approximate solution of diagnostic and therapeutic problems that concern both professions; if we are to be reciprocally helpful in developing a broader professional vision, and in acquiring a more comprehensive understanding of the present trend in medical and dental education and practice; and, finally, if we are to realize that the individual and collective efforts of the teacher, the research worker, and the practitioners of medicine and dentistry are proceeding, not in diverging lines, nor yet in parallel lines, but in lines that converge to a common point and that represent the consummated ideal of both professions, preventive medicine and dentistry.

We can appreciably add to the impetus of this movement by a more widespread acceptance of the fact that the knowledge of the one profession is complementary to that of the other and by the utilization of this knowledge in a more detailed and frequent contact of the two professions at the bedside, in the clinic and in open discussions.

I will present some evidence to illustrate the present trend so far as it affects the undergraduate of both professions and in what manner specialization in effort has been the inspiration to progress in both professions. By specialization, I refer to a definite, intelligent, and sustained endeavor by one who, fundamentally grounded in the principles of his profession, preferably with a background of a well ordered and carefully analyzed general experience, has the ability, the aptitude and the enthusiasm to concentrate all of his powers, analytical and constructive, for the more complete understanding of a more or less circumscribed field of medicine or surgery.

Specialization in medicine has existed for a longer period of time than has specialization in dentistry, and this specialization has played a most important role in the extension of medical knowledge. By the operation of individual thought in the laboratory and in the clinic, by scatterad observers over long periods of time, has resulted in large part the generalization and collective medical knowledge of today. From the same source we may expect the enlarged knowledge of tomorrow.

Dentistry is following the same cycle, and, like medicine, producing its practitioners, its research workers and its teachers. The last two classes, the research worker and the teacher, definitely link our two professions. The research workers in the two professions are actuated by the same desire, they have the same fundamental training and they are solving very similar problems, for they are primarily concerned in a study of the causation and prevention of disease. The teachers are primarily concerned in the dissemination of the methods adopted by the research workers and the results achieved by the practitioner in the alleviation and cure of disease.

If the practitioner, medical or dental, is to keep in touch with the advanced thought of his profession, if he is to give to his clientele the benefit of improved diagnostic methods and refined therapeutic procedures, he must establish some contact with the source from which this information continually flows.

My own experience in medical teaching and practice (for twenty-two years) has naturally led to a crystallization of opinion along this line. Not the least important of these conclusions has been a recognition—

*First*, of the deadening influence that follows a voluntary or involuntary professional isolation in the practice of medicine, whether in the heart of a congested city, or in the sparsely settled country districts;

*Second,* a corollary of the preceding, the stimulating effect of daily contact with fellow workers, and young enthusiasts, and the great benefit to be derived from a critical review of the weekly, monthly, or quarterly summaries of the achievements of fellow workers, or an account of the origination of researches in new fields;

*Third,* is the necessity for a co-ordination of thought and opinion in the allied realms of general medicine and its specialties: general surgery and its specialties; dentistry and its specialities, in the light of a more illuminating physiology, pathology, bio-chemistry and pharmacology.

If we follow the present trend of the educational movement in Medicine and Dentistry, we must be impressed by the similarity of purpose and identity of expression by the leaders in these great spheres.

For example, Eycleshymer, University of Illinois, in an address on "Liberalization in Medical Education" has this to say: "In human progress there are two fundamental processes which sometimes proceed equally, but usually one or the other is dominant. * * * These two processes are extension and consolidation. In the past medicine was largely restricted to the diseases of mankind. At present she recognizes the ultimate relationship of the diseases of plants and animals to those of mankind. In the near future she must take into consideration the diseases of metals: ultimately her domain will extend over both the organic and the inorganic world. * * * In the growth of knowledge in all of its special fields and great provinces as a whole the same two processes stand forth, specialization and generalization. The vitalizing factors in these are individual thought and collective thought. Greater men in medicine must come through great liberty in medical education. The practitioner of the future, either general or special, not only must measure up in self-reliance, responsibility and judgment to the practitioner of the past, but also must be better trained and more thoroughly imbued with the investigative spirit.

Acuteness in observation, precision in experimentation, and caution and judgment in deduction, are the essentials for the interpretation of disease.''

Now as a parallel idea the following excerpt is taken from an editorial in one of the dental journals. (The *Dental Cosmos*, August, 1922.)

''If one were to seek for the principal underlying cause of the revolutionary change that has led up to present day dental science and art * * * the germ theory of disease should rank first in importance. From the light thrown upon the etiology of dental caries by Miller to the present day knowledge of the bacteriopathology of the oral cavity and the causal relation of mouth infection to systemic disease, the science and art of dentistry have experienced a new birth * * * the objectives of dental practice are primarily vital rather than mechanical. The basis of dentistry is now biologic and its mechanical procedures are not ends in themselves. * * * The training of the latter day dental student must include a practical acquaintance with the broad underlying generalizations of science and the methods of reasoning by which these fundamentals have been evolved. In dental teaching there is needed a curriculum that will be educative as a whole, not merely informative.''

If these educational principles are desirable for the undergraduate body, their continued application is essential for us, who have left the doors of our Alma Mater, but who are still students attempting to acquire a much desired post-graduate culture while in the service of a jealous mistress.

Both professions in this state have worthy practitioners and eminent teachers; both professions for their highest devolopment need to produce more research workers. With vocational teachers in our state medical and dental colleges and teaching hospitals, with unlimited laboratory facilities and abundant clinical material, can we not feel reasonably assured that the day is not far distant when our undergraduate and graduate student body will feel the call to this higher professionalism and, by their efforts, place Virginia schools and the Virginia professions in the forefront of investigative work?

The progress of dental science has been mose closely watched and evaluated by your own leaders. The recognition of this progress by the medical profession was fairly sudden and coincident with the demonstration of the importance of oral sepsis.

I do not for a moment minimize the part played by our medical leaders in demonstrating the existence of oral sepsis and its relation to systemic conditions, but I would point out that the reaction of the medical mind, when it had once accepted as a possibility the importance of oral sepsis, varied somewhat according to its professional age and to the criteria the individual had assumed as necessary to prove the efficacy of any therapeutic procedure. The practical application of this principle of oral sepsis was possible only with the aid of the dental practitioner. Frequently the combination of the two, enthusiastic disciples of new thought, let credulity run away from judgment. In this connection we remember the acceptance by many of the profession of the role allotted the amoeba in the production of pyorrhea and the brief reign of glory enjoyed by emetine in the treatment of the condition. The sober consideration that followed the shattering of many ill-considered but optimistic prognoses and the realization that multiple foci of infection might exist tended to stabilize the medical and dental enthusiast on this engrossing subject.

The graduate of recent years, well trained in laboratory methods, in the theory of infection and immunity, is better aware of the possibility of multiple foci, other than the mouth, and also of the fact, that while foci of infection may originate and tend to perpetuate systemic disorders, yet, in many cases, secondary intoxication, metabolic rather than infectious, may assume the dominating role and perpetuate a vicious circle. It is in this type of case, for instance hypertrophic arthritis, that great disappointment has followed a failure to obtain relief after the surgical removal of one or more septic foci.

I doubt if there is any one subject on which there is such unanimity of opinion in the two professions, or any subject which has been more thoroughly discussed in all its ramifications than the subject of oral sepsis. Aside from its manifestations as a frank sepsis, we have had our attention directed to the role it may play in the essential anemias, achylia gastrica, certain forms of insanity, various arthritides, vascular and renal degenerations, and endocrine dyscrasias. The importance of oral prophylaxis and hygiene is so well established that it does not require further mention in this particular paper.

At this time, attention may be focussed not on the teeth as such, but on the mouth and all of its structures, mucous membrane, submucosa, the osseous tissue of the maxillae, the mandible, the teeth and the elveolar structure, the blood and lymph supply, the innervation; the functional ability of the mouth structures and jaw musculature, together with a consideration of developmental anomalies, acquired defects, the effect of traumata and bad habits and the modification in function that accompanies retrogressive tissue changes in the body, however, induced, and systemic states as reflected in the mouth.

This enumeration opens a field of far greater dimensions than that of oral sepsis. For the whole, obviously, is greater than any of its parts, no matter how important a fraction may be as compared to the whole. Oral sepsis is concerned with the principles of infection and immunity, with predisposing and active agents, with local and general tissue resistance. This larger field, in addition, is concerned with the findings of embryology, and the application of the principles and teachings of physiology and biochemistry. It emphasizes the interrelation of organ function to organ function as part of a general whole. It calls for as liberal a fundamental training on the part of the dentist as is now given to the medical man.—*Dental Digest.*

